# Iodine supply and thyroid function in women with gestational diabetes mellitus: a cohort study

**DOI:** 10.1530/EC-24-0295

**Published:** 2024-10-07

**Authors:** Hana Vítková, Kateřina Anderlová, Jan Krátký, Radovan Bílek, Drahomíra Springer, Felix Votava, Tomáš Brutvan, Adéla Krausová, Kristýna Žabková, Eliška Potluková, Jan Jiskra

**Affiliations:** 1 Third Department of Medicine, First Faculty of Medicine, Charles University and General University Hospital in Prague, Prague, Czech Republic; 2 Department of Gynaecology, Obstetrics and Neonatology, First Faculty of Medicine Charles University and General University Hospital in Prague, Prague, Czech Republic; 3 Institute of Endocrinology, Prague, Czech Republic; 4 Institute of Clinical Biochemistry and Laboratory Medicine, First Faculty of Medicine, Charles University and General University Hospital in Prague, Prague, Czech Republic; 5 Department of Children and Adolescents, Third Faculty of Medicine, Charles University and University Hospital Kralovske Vinohrady, Prague, Czech Republic; 6 University Center of Internal Medicine, Cantonal Hospital Baselland and University of Basel, Switzerland

**Keywords:** diabetes, iodine, metabolism, pregnancy, thyroid

## Abstract

**Introduction:**

Maternal urinary iodine concentration and blood neonatal thyroid-stimulating hormone (TSH) concentration reflect iodine status in pregnancy and serve as markers of iodine deficiency. As dietary measures in gestational diabetes mellitus (GDM) could affect iodine intake, our study aimed to investigate iodine supply in women with GDM compared to healthy pregnant women and to evaluate its relationship to maternal and neonatal thyroid function.

**Methods:**

Urinary iodine concentration (UIC) and serum TSH, free thyroxine (FT4), and autoantibodies against thyroid peroxidase (TPOAb) were analyzed in 195 women with GDM and 88 healthy pregnant women in the second trimester. Subsequently, neonatal TSH concentrations measured 72 h after delivery in a subgroup of 154 newborns (115 of mothers with GDM and 39 controls) from the national register were analyzed.

**Results:**

Median UIC was significantly lower in women with GDM compared to controls (89.50 µg/L vs. 150.05 µg/L; *P* < 0.001). Optimal iodine intake was found only in nine women with GDM (4.6%) and 33 healthy pregnant women (37.5%) (*P* < 0.001). Most pregnant women with GDM (88.7%) compared to one half of controls (50%) had iodine deficiency (*P* < 0.001). Although serum TSH and the prevalence of hypothyroidism (TSH > 4.0 mIU/L) were not different in both groups, hypothyroxinaemia was more prevalent in GDM compared to controls (12.3% vs 3.4%, *P* = 0.032). Consistently, neonatal TSH > 5.0 mIU/L indicating iodine deficiency, was found in 6 (5.2%) newborns of women with GDM as compared to none in controls. In women with GDM, the prevalence of perinatal complications was significantly lower in those who were taking dietary iodine supplements compared to those who were not (3/39 (7.69%) vs 46/156 (28.85%), *P* <0.001). In the multiple logistic and linear regression models in women with GDM, hypothyroxinaemia was associated with preterm births, and a negative association of serum FT4 and HbA1c was found.

**Conclusion:**

Iodine deficiency in pregnancy was more prevalent among women with GDM compared to healthy pregnant controls. Serum FT4 negatively correlated with HbA1c, and hypothyroxinaemia was associated with preterm births in women with GDM. Conversely, women with GDM who used dietary iodine supplements had a lower risk of perinatal complications.

## Introduction

Iodine is essential for thyroid hormone synthesis and normal child development. Iodine deficiency can manifest primarily during pregnancy and lactation, causing irreversible changes in the psychomotor and neurological development of the fetus and newborn. Iodine deficiency and autoimmune thyroiditis are the most common causes of maternal hypothyroidism and isolated hypothyroxinemia in pregnancy (1).

The World Health Organization’s (WHO) endorsement of salt iodization in 1952 as a countermeasure against iodine deficiency has been a cornerstone in public health policy. Data over the last two decades illustrate a pivotal shift: the proportion of countries meeting the criteria for adequate iodine intake surged from 67 in 2003 to 118 in 2020. This trend underscores the effectiveness of the WHO’s iodization strategy in mitigating iodine deficiency on a global scale (2, 3, 4). However, several studies have shown that the iodine supply for pregnant and lactating women remains inadequate, even in countries where the general population has achieved the optimal iodine supply. Therefore, the WHO recommends supplementation with 250 mcg of iodine daily during pregnancy and lactation.

There are several methods to evaluate the iodine supply of the population, including the measurement of urinary iodine concentration (UIC), examination of thyroid volume by ultrasound, measurement of maternal serum thyroglobulin concentration, or assessment of neonatal serum thyroid-stimulating hormone (TSH). Whereas thyroid volume and serum thyroglobulin indicate iodine nutrition over months or years, UIC and neonatal serum TSH reflect recent changes in iodine status and serve as sensitive markers of current iodine intake.

Since the 1990s, some studies have pointed to a possible relationship between thyroid dysfunction and/or thyroid autoimmunity and gestational diabetes mellitus (GDM) (5). Recently, Sitoris *et al.* (6) reported that thyroid autoimmunity in euthyroid women is associated with gestational diabetes in women older than 30 years.

Moreover, several studies have shown an increased risk of GDM in women with hypothyroidism diagnosed in the first trimester of pregnancy (7, 8, 9). Also, a higher prevalence of lower serum thyroxine (hypothyroxinaemia) regardless of TSH levels was found in pregnant women with diabetes mellitus as compared to controls (10). Finally, several complications during pregnancy and in newborns, including abortion, preterm birth, primary caesarean delivery, preeclampsia, fetal distress, and infant neurodevelopmental dysfunction, are more prevalent in women with thyroid dysfunction and GDM when compared to a healthy population (11, 12).

The objectives of our study were twofold: firstly, to compare iodine supply, thyroid function parameters, and thyroid antibodies between women with GDM and those without during the second trimester of pregnancy (in a cross-sectional analysis); and secondly, within the cohort of pregnant women with GDM, to evaluate the impact of these factors on perinatal outcomes, complications, and neonatal thyroid function.

## Patients and methods

### Patients

Between 2016 and 2017, 227 women newly diagnosed with GDM in screening with an oral glucose tolerance test (OGTT) performed in the second trimester of pregnancy, and 98 pregnant women with negative OGTT as a control group were included in the study. Forty-two women treated with levothyroxine were excluded from the analysis; therefore, 195 women with GDM and 88 controls were finally analyzed. OGTT was performed based on a standardized protocol using 75 g glucose orally. Cut-offs for positive OGTT were determined as any of the following: ≥ 5.1 mmol/L for fasting blood glucose repeatedly, ≥ 10.0 mmol/L at 60 minutes, ≥ 8.5 mmol/L at 120 min.

Fasting serum samples for measurement of concentrations of TSH, free thyroxine (FT4), and autoantibodies against thyroid peroxidase (TPOAb), and morning urine samples for measurement of iodine urine concentrations were collected, and all women completed a questionnaire focusing on a personal history of thyroid diseases, autoimmune and other diseases, medication, and iodine use during pregnancy. Women with GDM were followed, and data on diabetes compensation in the third trimester (glycated hemoglobin - HbA1c), childbirth and newborn outcomes and complications during pregnancy and after delivery were recorded.

Finally, neonatal TSH concentrations measured 72 h after delivery were recorded in 154 newborns (115 newborns of women with GDM and 39 controls) utilizing data from the national register of screening for congenital hypothyroidism.

The study protocol was approved by the Ethics Committee of the General University Hospital in Prague. All women included in the study signed a written informed consent.

### Biochemical assessment

Thyroid parameters (TSH, FT4, and TPOAb) were measured by chemiluminescence immunoassay on an ADVIA Centaur Analyzer (Siemens Healthcare Diagnostics Inc., Tarrytown, NY, USA). The reference interval for TSH in the second trimester of pregnancy was determined to be in the range of 0.5–4.0 mIU/L based on previous studies (13, 14).

As the FT4 immunoassay was changed during the study period, the reference range was different for women with GDM (11.5–22.7 pmol/L) and controls (10.0–22.7 pmol/L).

TPOAb concentrations higher than 60 kIU/L were considered positive, as stated by the producer.

Neonatal serum TSH was determined by immunofluorescence from a dry drop of blood taken on a screening filter paper card. According to Hnikova and Rysava, a prevalence of elevated neonatal TSH levels exceeding 5 mIU/L in more than 3% of the population would suggest significant iodine deficiency (15, 16).

UIC were measured by absorption spectrophotometry after alkalization and demineralization. In pregnant women, UIC of 150–249 µg/L corresponds to an optimal iodine supply (17).

### Statistics

GraphPad Prism version 8 (GraphPad Software, San Diego, CA, USA) and SigmaStat statistical software (Jandel Corporation, San Jose, California, USA) were used for statistical processing. The *t*-test, Mann–Whitney *U* test, Wilcoxon test, Kruskal–Wallis test, Chi-square test, and Fisher’s exact test were used to compare patients and controls. Spearman’s rank correlation coefficient served to evaluate the significance of the correlations. A multiple linear and logistic regression model was developed to assess the effect of UIC on thyroid parameters, the effect of UIC and thyroid parameters on maternal HbA1c, birth complications and neonatal parameters, and the effect of UIC, thyroid parameters, and HbA1c on neonatal complications and neonatal parameters. A *P* value of <0.05 was considered significant.

## Results

The main results of the cross-sectional part of the study are summarized in Table 1.

**Table 1:** Comparison of UIC and serum thyroid parameters in mothers with gestational diabetes mellitus and controls.

	GDM	Controls	*P*
Number	195	88	
Age^1^	33 (28–37)	33 (30–36)	0.796
Body mass index^1^	24 (21–29)	22 (20–24.5)	0.085
Users of dietary iodine supplements^2^	39 (20.00%)	36 (40.91%)	< 0.001
UIC^1^	89.50 (68.30–111.80)	150.05 (103.35–211.90)	< 0.001
UIC^1^ in non-users of dietary iodine supplements	89.10 (68.35–112.00) *n* = 156	145.45 (82.95–194.65) *n* = 52	< 0.001
UIC 150–499 ug/L (optimal)^2^	22 (11.28%)	42 (47.73%)	< 0.001
UIC < 150 ug/L^2^	173 (88.72%)	44 (50.0%)	< 0.001
UIC < 100 ug/L^2^	120 (61.54%)	20 (22.73%)	< 0.001
UIC 50–149 ug/L^2^	154 (78.97%)	38 (43.18%)	< 0.001
UIC 20–49 ug/L^2^	19 (9.74%)	6 (9.89%)	0.861
UIC < 20 ug/L^2^	0	0	–
UIC ≥ 500 ug/L^2^	0	2 (2.23%)	–
TSH^1^	1.94 (1.41–2.66)	2.19 (1.57–2.69)	0.124
FT4^1^	13.03 ± 1.75	12.60 ± 1.65	0.102
FT3^1^	4.1 (3.90–4.40)	4.1 (3.9–4.37)	0.786
TPOAb^1^	44.0 (36.00–52.18)	33.0 (28.00–43.50)	< 0.001
Positive TPOAb^2^	23 (11.80%)	8 (9.09%)	0.639
Hypothyroxinemia^2,3^	24 (12.31%)	3 (3.41%)	0.032
TSH > –4.0 mU/L^2^	14 (7.18%)	5 (5.68%)	0.834
Neonatal TSH > 5 mU/L^2^	6 (3.90%) *n* = 115	0 *n* = 39	0.329

^1^expressed as median (upper quartile – lower quartile)

^2^expressed as a number (%)

^3^as the FT4 immunoassay was changed during the study period, the reference range was different for women with GDM (11.5–22.7 pmol/l) and controls (10.0–22.7 pmol/l)

FT4, free thyroxine (pmol/L); FT3, free thyroxine (pmol/L); GDM, gestational diabetes mellitus; TPOAb, antibodies against thyroid peroxidase (IU/L); TSH, thyroid stimulating hormone (mU/L); UIC, urinary iodine concentration (ug/L).

### Urinary iodine concentrations

Median UIC was significantly lower in women with GDM compared to controls (89.50 µg/L vs 150.05 µg/L; *P* < 0.001). Similar results were obtained when women taking dietary supplements containing iodine were excluded from the analysis (89.10 µg/L vs 145.45 µg/L, *P* < 0.001) (Table 1). Optimal iodine supply (UIC 150–499 µg/L) was found only in nine women with GDM (4.62%) as compared to 33 (37.5%) controls (*P* < 0.001). Mild iodine deficiency (UIC 50–149 µg/L) was found in 154 (78.97%) women with GDM, as compared to 38 (43.18%) controls (*P* < 0.001). Moderate iodine deficiency (UIC 20–49 µg/L) was found in less than 10% of pregnant women in both groups, and severe iodine deficiency did not occur among the examined women. In contrast, slightly excessive iodine saturation (UIC 250–499 µg/L) was present in 13 women with GDM and nine women in the control group (6.67% vs 10.23%; *P* = 0.075), and excessively increased iodine saturation (UIC ≥ 500 µg/L) was found only in two controls (2.23%) (Table 1).

### Dietary supplements and their influence on urinary iodine concentration

When analyzed collectively (both with and without GDM), pregnant women who had been taking dietary supplements containing iodine (n = 75) exhibited a significantly higher median UIC compared to those who had not (n = 208) (115.2 vs 94.85 µg/L, *P* = 0.026). Although not significant, this difference was more expressed in the control group, whereas in women with GDM, the UIC was not significantly affected by iodine supplements (Table 2).

**Table 2:** TSH and FT4 in relation to different UIC.

	Number^1^	TSH	FT4
UIC < 50 ug/L	25	1.67 (1.26–2.26)	13.07 ± 1.59
UIC 50–149 ug/L	192	2.02 (1.45–2.66)	13.11 ± 1.68
UIC 150–249 ug/L	42	2.11 (1.49–2.80)	12.36 ± 1.75
UIC ≥ –250 ug/L^2^	24	2.35 (1.70–3.22)	13.21 ± 1.51
*P* (ANOVA)		0.097	0.061

^1^women with GDM and controls were evaluated together in this sub-analysis.

^2^since there were only two women in the group with UIC ≥ 499 ug/L, they were evaluated together with the 250–499 ug/L group

FT4’ free thyroxine (pmol/L); TSH’ thyroid stimulating hormone (mU/L); UIC, urinary iodine concentration (ug/L), *P,P* value.

### Relationship of iodine supply and serum thyroid biochemical tests

Serum TSH concentrations and prevalence of TSH elevation >4 mIU/L did not differ between women with GDM and controls (Table 1). In contrast, hypothyroxinemia was found in 23 women with GDM and only three controls (12.31% vs 3.41%, *P* = 0.032) (Table 1). The concurrent elevation of serum TSH was demonstrated in only two women with GDM and no women in the control group. Serum FT3 concentrations and prevalence of positive TPOAb did not significantly differ between women with GDM and controls. Similarly, serum TSH and FT4 concentrations did not significantly differ across the groups with different UIC (women with GDM and controls were evaluated together in this sub-analysis) (Table 3).

**Table 3:** Urinary iodine concentrations in relation to use of dietary supplements with iodine.

	Users of dietary iodine supplements (*n* = 75)	Non-users of dietary iodine supplements (*n* = 208)	*P*
GDM (*n* = 195)	89.9 (69.55–110.15)	89.1 (68.35–112.0)	0.802
Controls (*n* = 88)	183.95 (124.65–215.95)	145.45 (82.95–194.65)	0.071
All women (*n* = 283)	115.2 (80.743–193.83)	94.85 (71.6–124.05)	0.026

Expressed as median (upper quartile – lower quartile)

GDM, gestational diabetes mellitus.

### Neonatal thyroid-stimulating hormone concentrations

Neonatal TSH concentrations were available from 154 newborns (115 of women with GDM and 39 controls). Six newborns of women with GDM (5.22%) had neonatal TSH concentrations >5 mIU/L, indicating significant iodine deficiency. On the contrary, no case of neonatal TSH >5 mIU/L was found in newborns of controls (n = 39). However, due to the small number of cases with neonatal TSH elevation, the difference was not significant. In the multiple linear and logistic regression models, we found no significant independent predictor of neonatal TSH in pregnant women with GDM (Tables 4 and 5).

**Table 4:** Multiple linear regression model in women with GDM.

Independent variables	Dependent variables							
	Mother’s FT4	Mother’s HbA1c (*n* = 79)	Neonatal TSH (*n* = 115)	Neonatal Apgar score	Birth weight	Birth length	Head crf.	Trunk crf.
Mother’s age	NS	NS	NS	NS	NS	NS	NS	NS
Mother’s weight	NS	NS	NS	NS	25.063 (6.91) *P* = 0.02	NS	NS	NS
Mother’s UIC	NS	NS	NS	NS	NS	NS	NS	NS
Mother’s TSH	NS	NS	NS	NS	NS	NS	NS	NS
Mother’s FT4	–	−1.491 (0.517) *P* = 0.007	NS	NS	NS	NS	NS	NS
Mother’s FT3	1.525 (0.673) *P* = 0.03	NS	NS	NS	602.263 (243.25) *P* = 0.026	4.165 (1.546) *P* = 0.017	NS	NS
Mother’s TPOAb	NS	NS	NS	NS	NS	NS	NS	NS
Mother’s HbA1c (*N*=79)	–	–	NS	NS	58.072 (22.22) *P* = 0.02	NS	NS	NS

Data are expressed as a coefficient (standard error).

crf, circumference; FT4, free thyroxine (pmol/L), FT3, free triiodothyronine (pmol/L); GDM, gestational diabetes mellitus; HbA1c: glycated haemoglobin (mmol/moL); P, level of significance; TSH: thyroid-stimulating hormone (mU/L) UIC, urinary iodine concentration (ug/L); TPOAb, antibodies against thyroid peroxidase (IU/L).

**Table 5:** Multiple logistic regression model in women with GDM.

	Dependent variables					
	Neonatal TSH >5.0 mU/L	Preterm birth	Low birth weight	Fetal hypothrophy	Fetal hyperthrophy	Birth complication
Iodine supplements users	NS	NS	NS	NS	NS	−0.242 (0.058–1.011) *P* = 0.05
UIC < 150 ug/L	NS	NS	NS	NS	NS	NS
TSH > 3.67 mU/L	NS	NS	NS	NS	NS	NS
FT4 < 11.5 pmol/L	NS	12.498 (1.126–138.761) *P* = 0.04)	NS	NS	NS	NS
Positive TPOAb	NS	NS	NS	NS	NS	NS
Mother’s HbA1c > 48	NS	NS	NS	NS	NS	NS
Parous women	NS	NS	NS	NS	NS	NS
History of abortion	NS	NS	NS	NS	NS	NS
IVF	NS	NS	NS	NS	NS	NS

Data are expressed as odds ratio (95% confidence interval).

GDM, gestational diabetes mellitus, HbA1c, glycated haemoglobin (mmol/moL); IVF, in vitro fertilization; LT4, levothyroxine; P, P value; TPOAb, antibodies against thyroid peroxidase (IU/L); TSH, thyroid stimulating hormone (mU/L); UIC, urinary iodine concentration (ug/L).

### Relationship of UIC and thyroid biochemical parameters to HbA1c and perinatal outcomes in women with GDM

In women with GDM, the prevalence of perinatal complications (acute fetal hypoxia, pathological cardiotocogram, fetal dystocia, fetal macrosomia, polyhydramnios, turbid amniotic fluid, placenta praevia, non-progressive labor, preterm premature rupture of membranes, episiotomy) was significantly lower in those who were taking dietary iodine supplements compared to those who were not (3/39 (7.69%) vs 46/156 (28.85%), *P* < 0.001). Consistently, the use of dietary iodine supplements was slightly associated with a lower risk of perinatal complications in a multiple logistic regression model (at the limit of statistical significance) (Table 5). Finally, in the multiple logistic regression model, hypothyroxinaemia was associated with preterm birth in women with GDM (Table 5).

**Figure 1:**
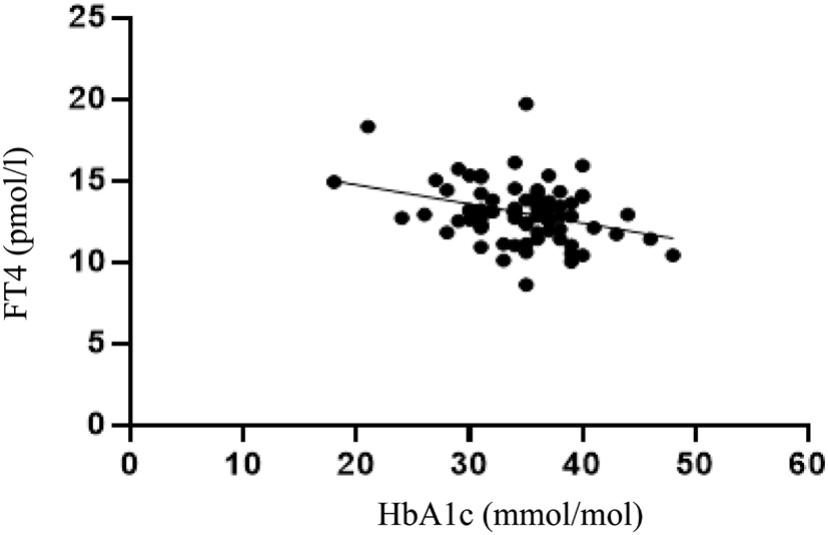
The negative association of FT4 and HbA1c in women with gestational diabetes mellitus in a linear regression analysis (coefficient −0.926, standard error 0.302, *P* = 0.003, *n* = 79). FT4: free thyroxine; HbA1c: glycated haemoglobin.

In the multiple linear regression model (Table 4), a negative association of FT4 with HbA1c (*P* = 0.007) was found, as well as positive associations of FT3 with birth weight (*P* = 0.026), FT3 with birth length (*P* = 0.017), and HbA1c with birth weight (*P* = 0.02). In linear regression analysis, only the strong negative association of FT4 with HbA1c remained significant (Coefficient −0.926, standard error 0.302, *P* = 0.003, *n* = 79) (Fig. 1). In multiple regression analysis, BMI was not an independent predictor of UIC in our study.

## Discussion

Our findings reveal that, within an iodine-sufficient region, pregnant women diagnosed with GDM exhibit significantly higher rates of iodine deficiency – as determined by UIC – compared to control women without GDM (88.7% vs 50%, respectively). Our data add evidence to the linkage between iodine deficiency during pregnancy and GDM. Considering that GDM is prevalent in roughly 5% of all pregnancies and is associated with serious risks such as congenital anomalies, spontaneous abortions, and higher incidences of type 2 diabetes and obesity in both the mother and the offspring, identifying iodine deficiency as a potentially preventable risk factor for GDM could have profound implications for health policies (18). The association between iodine deficiency and diabetes mellitus, including GDM, is intriguing. It has been shown that people diagnosed with type 2 diabetes had lower UIC compared to the control groups and that the UICs were inversely linked with the plasma insulin levels and the Homeostatic Model Assessment of Insulin Resistance (HOMA-IR) index (19, 20, 21). Moreover, higher concentrations of placental iodine are linked with a lower incidence of GDM, and a lower placental iodine load is associated with an altered plasma insulin concentration, HOMA-IR index, and β-cell activity (22). The association between iodine deficiency and diabetes may be mediated through insulin, given that research has identified a correlation between iodine deficiency and insulin resistance (22). In addition, a large epidemiologic study (TIDE) showed that obese women had significantly lower UIC compared to both themselves after bariatric surgery and normal-weight women, and the incidence of central obesity was lower when UIC was 300 μg/L or higher (23, 24). Moreover, a negative association of iodine deficiency with BMI was found (25). In contrast, BMI was not an independent predictor of UIC in our study.

It may be perceived that the issue of iodine deficiency has been largely addressed through the implementation of global salt iodization strategies (26). However, pregnant and lactating women in many countries still face iodine insufficiency. Studies reveal inadequate iodine levels in pregnant women in China (27), across Europe (28), and the United States (29), despite generally adequate national iodine intake. Even in the Czech Republic, where salt has been iodized since the 1950s, a majority of pregnant women showed iodine deficiency, highlighting the ongoing challenge in ensuring sufficient iodine during pregnancy (30).

In a population-based case-control study in Finland, low serum levels of iodide and thyroid function in early pregnancy were not associated with an increased risk of GDM (31), however, a higher serum iodide was positively associated with preterm birth (32). To our knowledge, apart from the study by Lindorfer *et al.* (33), our current study is only the second in the world looking at iodine intake in pregnant women with GDM assessed by UIC. In pregnant women with GDM, we found an identical median UIC to that of Lindorfer *et al.* (89.5 µg/L) (33). However, our control women without GDM had significantly higher UIC (150.1 µg/L) compared to his findings (82.9 µg/L) (33). This corresponds to inadequate iodine supply in 88.7% of pregnant women with GDM and 50.0% of pregnant women without GDM. These findings are consistent with our previous study indicating that women after spontaneous abortions were almost twice as likely to suffer from mild iodine deficiency and had a lower median UIC compared to age-matched controls (34).

Contrary to the recommendation of the European Thyroid Association (35), only 20% of women with GDM and 40.9% of healthy controls reported using iodine supplements in our study. Supplementation leads to higher UIC, corresponding to the findings of Lindorfer *et al.* (33). In our study, this was only apparent when both groups (with and without GDM) were analyzed together – likely a result of the small sample size. Consistent with our previous results (34) and other studies (36, 37), we found no relationship between iodine supply, the degree of iodine deficiency, and thyroid function. Among women with GDM, we found no significant difference in UIC between those who used iodine supplements and those who did not. However, the GDM group exhibited a significantly lower median UIC compared to the control group, a difference that persisted even after excluding users of iodine supplements from the analysis. Moreover, we found that in women with GDM, the prevalence of perinatal complications was significantly lower in those who were taking dietary iodine supplements compared to those who were not. We cannot quite explain the discrepancy between the missing increase in UIC in women taking supplements and such clinical findings like the decreased prevalence of perinatal complications in these women. We can only speculate that the measurement of UIC might not adequately reflect the true iodine intake due to its high variability and dependence on the actual intake on the given day, but the overall higher iodine intake attenuated the occurrence of perinatal complications. Due to the relatively small sample size, we should interpret these results with caution. Nevertheless, they are interesting and suggest that supplementation with iodine might decrease the risk of perinatal complications.

Research on the link between thyroid dysfunction during pregnancy and GDM yields mixed findings. Studies like those by Stohl *et al.* (38) and Oguz *et al.* (39) report varying GDM rates among hypothyroid and hyperthyroid women, while Tudela *et al.* (7) suggest elevated TSH in early pregnancy might predict GDM risk. Despite some studies supporting these connections (8, 9, 40, 41, 42, 43), others find no relationship (44, 45, 46). The Thr92Ala deiodinase D2 polymorphism, obesity-related TSH elevation, and dietary effects on deiodinase activity are potential factors (47, 48). Yet, Mannisto *et al.* (40) observed that levothyroxine treatment does not alter GDM risk, highlighting the complex nature of these relationships and the need for more research.

The prevalence of isolated hypothyroxinemia in pregnancy varies between 1.3% and 25.4% and could be caused by iodine deficiency (1). Although our study did not reveal an association between iodine deficiency and serum TSH concentration, we observed a 3.6 fold higher prevalence of hypothyroxinemia in the iodine-deficient women with GDM compared to controls. Furthermore, linear regression analysis showed a strong negative association between FT4 levels and HbA1c in pregnant women with GDM. This is consistent with the previously reported higher prevalence of hypothyroxinemia in women with GDM regardless of TSH concentration (10) and the higher risk of occurrence of GDM in the second trimester in women with hypothyroxinemia (odds ratio 1.89, 95% CI 1.26–2.84) (47). The impact of isolated hypothyroxinemia on the course of pregnancy, childbirth, and the perinatal period remains unclear. Some studies (49, 50) report no significant effects on pregnancy complications or fetal development, while others (51) link it to fetal issues and preterm birth. Our findings suggest a marginal association between hypothyroxinemia and preterm birth in women with GDM.

Consistent with previous studies showing no relationship between positive thyroid antibodies and GDM risk (40, 41, 44, 46), we found no difference in the prevalence of positive TPOAb antibodies in the GDM group and controls.

As neonatal TSH assessed for the purpose of screening for neonatal hypothyroidism may reveal iodine deficiency (52), we used it as an additional marker of the iodine supply for pregnant women with GDM. Generally, the prevalence of neonatal TSH elevated >5 mIU/L in more than 3% of newborns indicates significant iodine deficiency in the population (15, 16). In the Czech Republic, the proportion of newborns with iodine deficiency reached 3.8% in 2019 and rose to 4.8% in 2020 (53). Compared to these data, the 5.22% prevalence of elevated neonatal TSH in newborns from mothers with GDM in our study corroborates our findings, indicating a more pronounced iodine deficiency during pregnancy in women with GDM compared to controls.

Our study presents several limitations. First, the assessment of iodine deficiency via UIC could be more accurate if renal function, particularly glomerular filtration rates, were considered. However, these aspects are rarely included in similar research due to their high demand for resources. Our analysis compared young women newly diagnosed with GDM, without chronic diabetes complications, to a control group of pregnant women, leading to an assumption of similar glomerular filtration rates between the groups. Secondly, our study did not include measurements of serum thyroglobulin, a marker for iodine deficiency in pregnancy highlighted by recent studies (54, 55), indicating a gap that future research should address. Thirdly, the accuracy of FT4 evaluation during pregnancy via immunoassays is affected by changes in distribution volume and binding proteins, resulting in difficulties comparing study outcomes due to significant interindividual and week-to-week variability. Additionally, the method for measuring FT4 was altered during our study, leading to different reference ranges and complicating result interpretation. Future studies should employ liquid chromatography-tandem mass spectrometry (LC/MS-MS) for more precise analysis of FT4. Lastly, the small sample size of our study complicates the interpretation of results, limiting the generalizability of our findings.

In conclusion, our study highlights a significant prevalence of iodine deficiency among pregnant women with GDM, in contrast to a control group of healthy pregnant women. We observed a negative correlation between serum FT4 levels and HbA1c, and hypothyroxinaemia was associated with preterm births in women with GDM. Our findings suggest that dietary iodine supplementation in pregnant women with GDM may confer a protective benefit, evidenced by a lower incidence of perinatal complications. These results underscore the importance of adequate iodine nutrition during pregnancy, particularly among those diagnosed with GDM, to mitigate adverse pregnancy outcomes. Yet, prospective randomized studies are required for definitive conclusions.

## Declaration of interest

The authors declare that there is no conflict of interest that could be perceived as prejudicing the impartiality of the research reported.

## Funding

This research was funded by the project MH CZ – DRO (General University Hospital in Prague – VFN, 00064165).
